# Supplementary material for: Integrated Methylome and Transcriptome Analysis between the CMS-D2 Line ZBA and Its Maintainer Line ZB in Upland Cotton
Source: Int J Mol Sci. 2019 Dec 2;20(23):6070. doi: 10.3390/ijms20236070 (PMC6928835; doi:10.3390/ijms20236070)
Supplement: Supplementary file 1 [file ijms-20-06070-s001.zip › Figures. S1-S8.pdf]

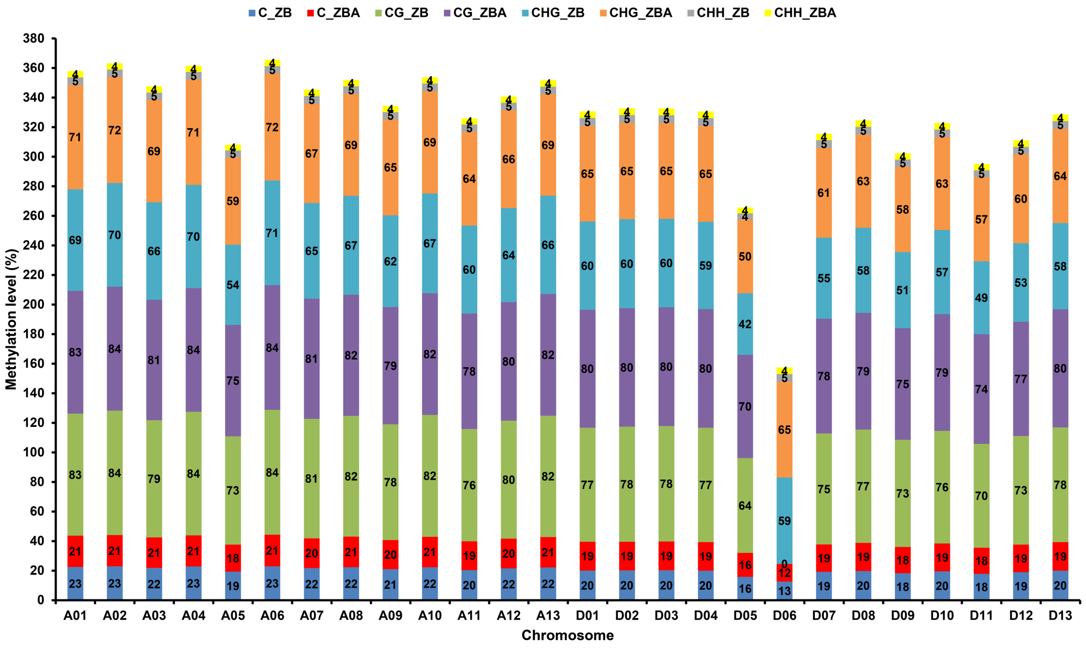

Figure S1. Levels of C, CG, CHG, and CHH methylation in 26 chromosomes. The X-axis represents all 26 chromosomes of upland cotton; the Y-axis represents methylation levels (MLs) in different sequence contexts.

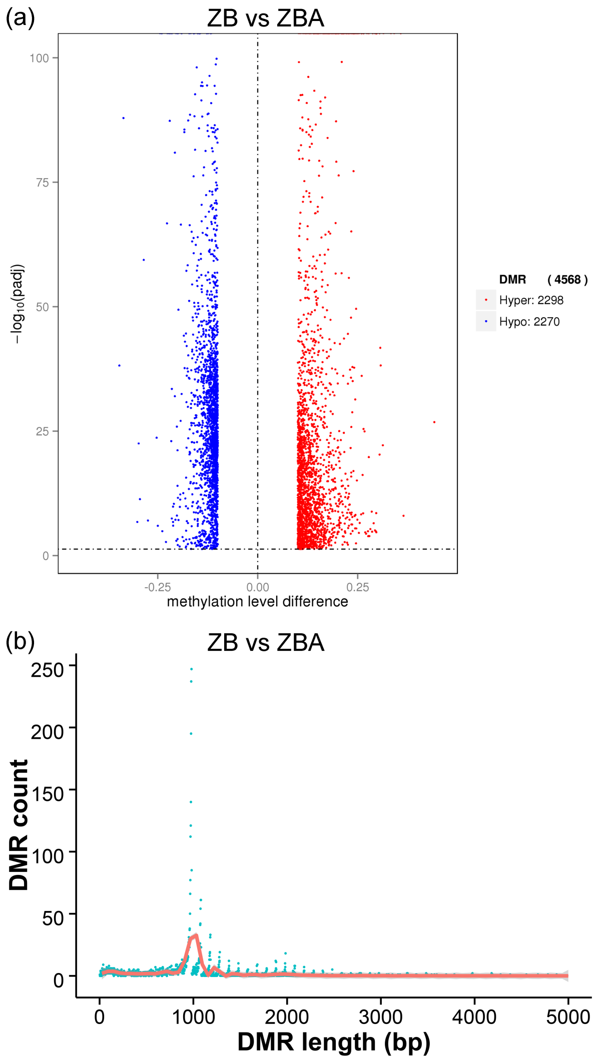

Figure S2. Volcano map of identified differentially methylated regions (DMRs) (**a**) and their length distribution (**b**) in ZB versus ZBA. In the volcano map, each dot signifies one DMR. Among them, the red and blue dots represent hyper- and hypomethylated DMRs, respectively. The X-axis represents the ML difference; the Y-axis represents the opposite number of log_{10} value of the *P*-value.

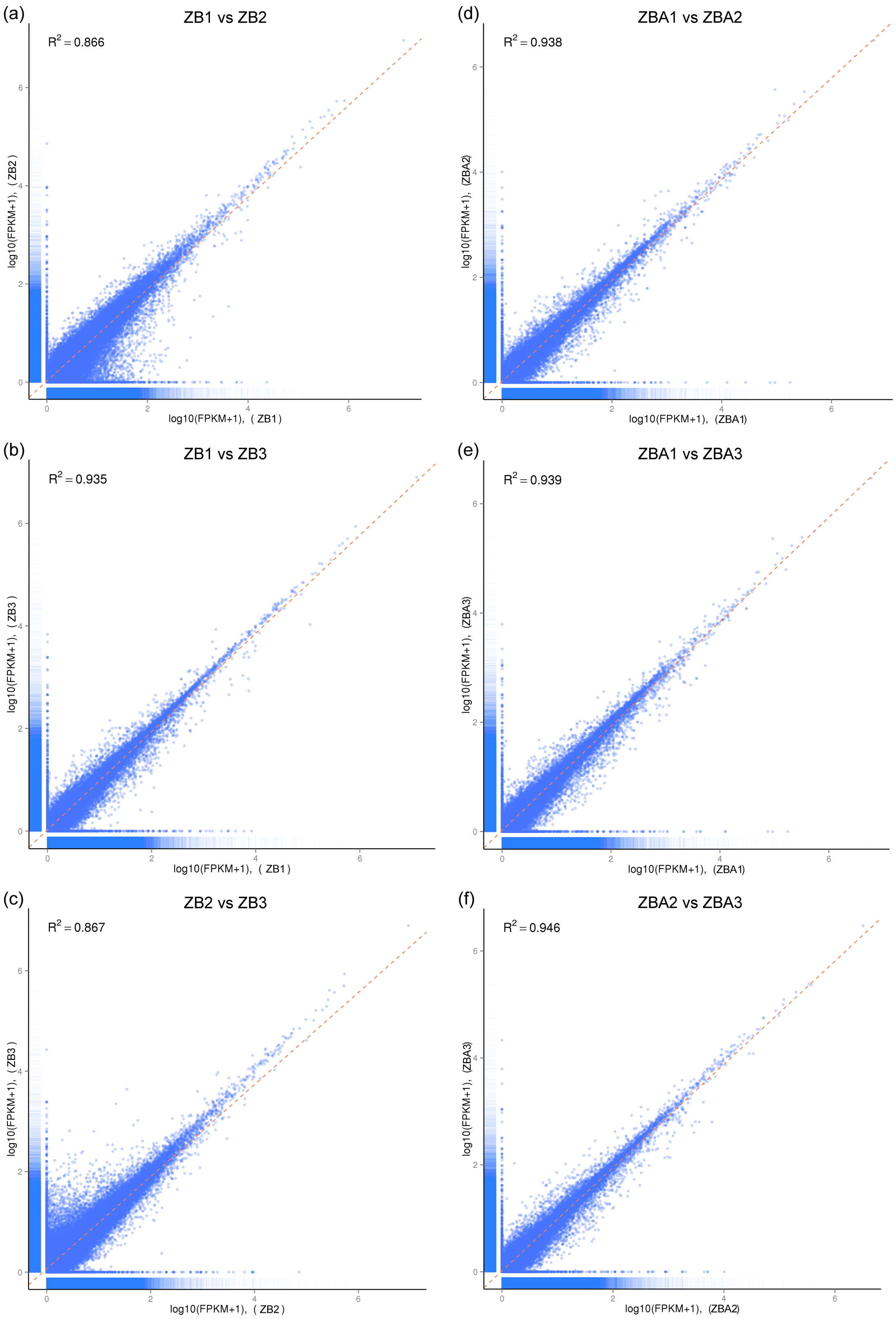

Figure S3. Evaluation of RNA sequencing data quality. (**a**) to (**c**) Pearson's Correlation coefficient testing the ZB dataset. (**d**) to (**f**) Pearson's Correlation coefficient testing the ZBA dataset.

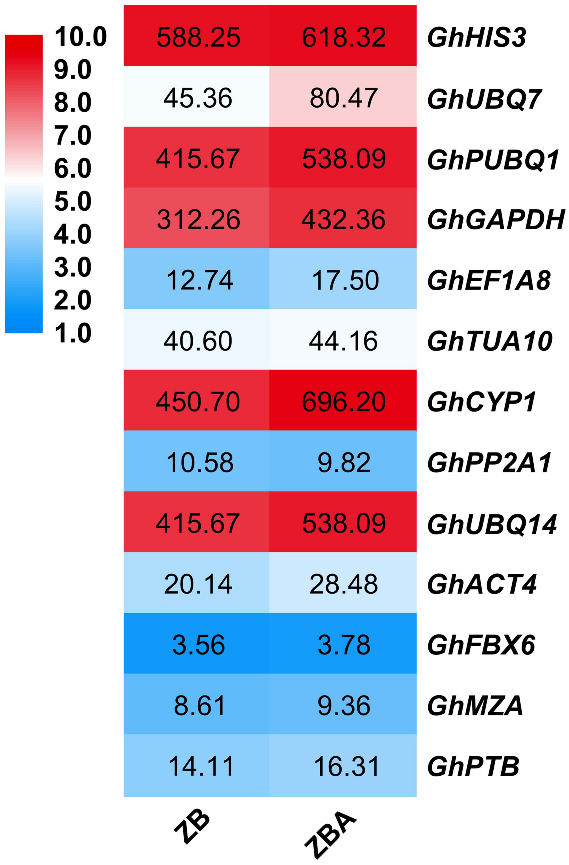

Figure S4. Heatmap of 13 selected cotton reference (housekeeping) genes with their respective FPKM values in ZB and ZBA. Log_2 expression (FPKM) values are used for the analysis and negative values are set to zero.

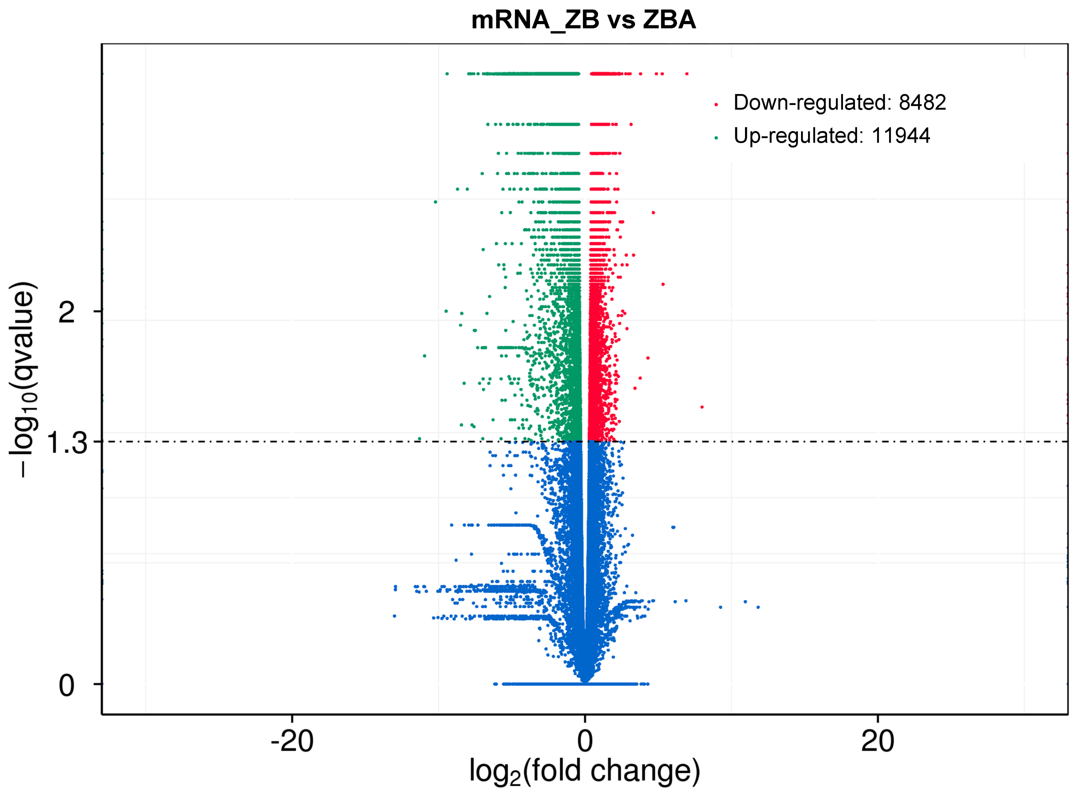

Figure S5. Numbers of differentially expressed genes (DEGs) in ZB versus ZBA. The X-axis is the $\log_2$ value of fold change and the Y-axis is the opposite number of $\log_{10}$ value of the *q*-value (adjusted *P*-value). Each dot represents one gene. Among them, the green and red dots represent up- and down-regulated genes, respectively, and the blue dots represent genes without differential expression.

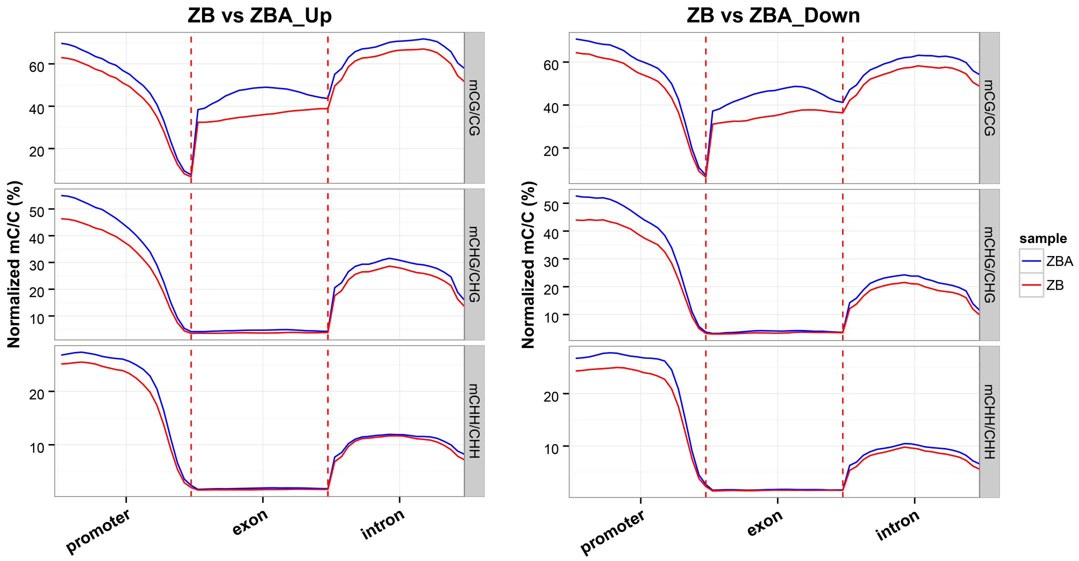

Figure S6. Comparative analysis of methylation densities of C-sites in different gene regions of the up- or downregulated DEGs, including promoter, exon, and intron regions.

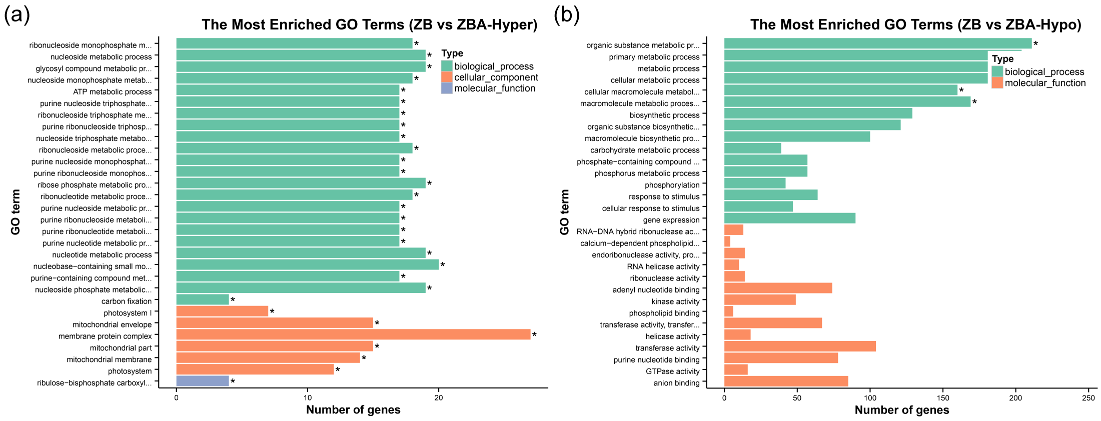

Figure S7. Gene ontology (GO) enrichment analysis of hyper- (**a**) or hypomethylated (**b**) DEGs in ZB versus ZBA. The bars followed by an asterisk are some significantly enriched GO terms.

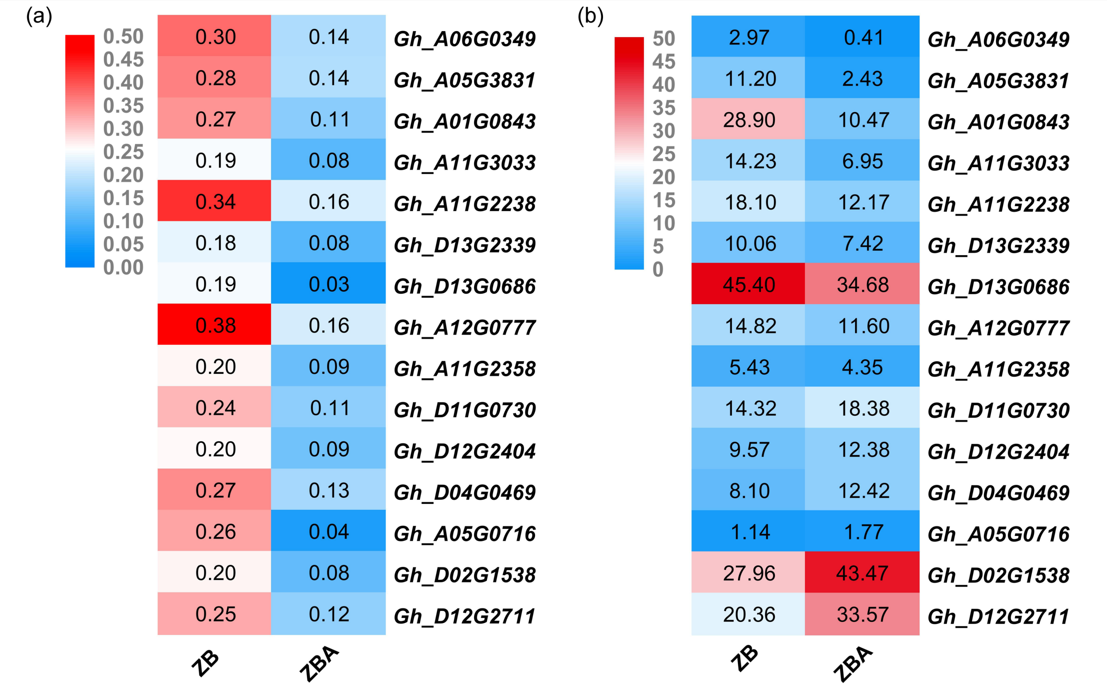

Figure S8. Heatmaps showing the MLs (**a**) and the expression levels (i.e., FPKM values) (**b**) of hyper-DEGs involved in the "mitochondrial envelope" term in ZB versus ZBA.
